# Cryo‐Electron Tomography of *Toxoplasma gondii* Indicates That the Conoid Fiber May Be Derived from Microtubules

**DOI:** 10.1002/advs.202206595

**Published:** 2023-02-25

**Authors:** Zhixun Li, Wenjing Du, Jiong Yang, De‐Hua Lai, Zhao‐Rong Lun, Qiang Guo

**Affiliations:** ^1^ State Key Laboratory of Protein and Plant Gene Research, Peking‐Tsinghua Center for Life Sciences, Academy for Advanced Interdisciplinary Studies, School of Life Sciences Peking University Beijing 100871 P. R. China; ^2^ MOE Key Laboratory of Gene Function and Regulation, State Key Laboratory of Biocontrol, School of Life Sciences Sun Yat‐Sen University Guangzhou 510275 P. R. China; ^3^ Changping Laboratory Yard 28, Science Park Road Beijing 102206 P. R. China

**Keywords:** Apicomplexa, conoid fibers, cryo‐electron tomography, endodyogeny, microtubules, tachyzoites

## Abstract

*Toxoplasma gondii (T. gondii)* is the causative agent of toxoplasmosis and can infect numerous warm‐blooded animals. An improved understanding of the fine structure of this parasite can help elucidate its replication mechanism. Previous studies have resolved the ultrastructure of the cytoskeleton using purified samples, which eliminates their cellular context. Here the application of cryo‐electron tomography to visualize *T. gondii* tachyzoites in their native state is reported. The fine structure and cellular distribution of the cytoskeleton are resolved and analyzed at nanometer resolution. Additionally, the tachyzoite structural characteristics are annotated during its endodyogeny for the first time. By comparing the structural features in mature tachyzoites and their daughter buds, it is proposed that the conoid fiber of the Apicomplexa originates from microtubules. This work represents the detailed molecular anatomy of *T. gondii*, particularly during the budding replication stage of tachyzoite, and provides a reference for further studies of this fascinating organism.

## Graphical abstract

This study utilized advanced cryo‐ET technology to provide a detailed molecular anatomy of *T. gondii*, with a particular focus on the budding endodyogeny stage of tachyzoites. In addition to confirming previous research, this study uncovered significant new features. Based on these findings, the study proposes that the conoid fiber, a critical component of the conoid, may have microtubule origins.

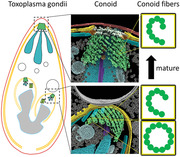

## 1 Introduction

*Toxoplasma gondii (T. gondii)* is one of the most successful parasites and can infect most warm‐blooded animals.^[^ ^1^ ^]^ More than one‐third of the world's population is estimated to be infected with this parasite.^[^ ^2^ ^]^ *T. gondii* infection is generally asymptomatic in people with normal immunity. However, it can be fatal to infants and immunosuppressed patients, such as organ transplant recipients and patients with AIDS or cancer.^[^ ^3^ ^]^

The life cycle of this parasite includes three infective stages: tachyzoites, bradyzoites (in the cysts), and sporozoites (in the oocysts). Tachyzoite is one of the asexual stages, during which *T. gondii* can move around the host cells and quickly reproduce once it has invaded.^[^ ^4^ ^]^ Tachyzoite is also the critical stage for pathogenesis in the host. Conventional electron microscope studies showed that an intact tachyzoite has a bow shape and possesses typical eukaryotic organelles.^[^ ^5^ ^]^ The pellicle of this parasite includes the plasma membrane (PM) and the inner membrane complex (IMC), which is comprised of flattened double‐membrane vesicles.^[^ ^6^ ^]^ Tachyzoites possess unique scaffold assemblies in addition to typical cytoskeleton structures, including subpellicular microtubules (SPMTs), intraconoidal microtubules (ICMTs), a conoid, and an apical polar ring (APR). The SPMTs are underneath the IMC and cover approximately two‐thirds of the tachyzoite. Evidence indicates that SPMTs are essential to the maintenance of cell shape.^[^ ^7^ ^]^ The conoid is a funnel‐shaped structure in the apical part of the tachyzoite; this structure is crucial for motility and changes from a “retracted” state to a “protruded” state during host cell invasion.^[^ ^8^ ^]^ However, little is known about the origin of this structure. It was observed that one conoid contains two parallel ICMTs with the ICMTs extending from the conoid in a typical fashion.^[^ ^7a^ ^]^ One of these ICMTs was found to be attached by several vesicles relevant to intracellular transport.^[^ ^7^, ^9^ ^]^ Each of these components in the assembly is essential for the life‐sustaining functions of tachyzoites and may potentially be used as drug targets against *T. gondii*.^[^ ^10^ ^]^ High‐resolution structures of the SPMT and conoid were recently resolved using cryo‐transmission electron microscopy;^[^ ^11^ ^]^ however, this approach required the isolation of the structures from the cells first. The obvious disadvantage of this “divide and conquer” approach eliminated the contextual information that is critical for understanding the function of these structures.

The asexual reproduction of *T. gondii* is called endodyogeny, which results in two new daughter buds in the cytoplasm of the mother cell. During this process, the nuclear envelope is kept intact^[^ ^12^ ^]^ and centrosomes are essential.^[^ ^13^ ^]^ Previous studies have shown that the daughter buds lack a PM but possess their own IMC, SPMTs, conoid, and ICMTs.^[^ ^5^, ^14^ ^]^ Impaired microtubules in tachyzoites lead to abnormal nuclear division during budding,^[^ ^7b^ ^]^ which indicates that the cytoskeleton plays a crucial function in endodyogeny. However, little is known about the molecular mechanism of endodyogeny, due in part to the lack of precise structural information.

Cryo‐electron tomography (cryo‐ET) is a powerful technique that can depict the subcellular details of vitrified samples in a 3D space at nanometer resolution under near physiological conditions. Furthermore, with the help of segmentation and subtomogram averaging, the fine structures and spatial distributions of macromolecules can be determined within their native infecting environment.^[^ ^15^ ^]^ Here, we report the molecular anatomy of the *T. gondii* tachyzoite and the daughter buds using cryo‐ET, with a particular focus on the cytoskeleton components.

## 2 Results and Discussion

### 2.1 Cryo‐ET Reveals the 3D Structure of *T. gondii* Tachyzoites at the Nanoscale

The life cycle of *T. gondii* has three infectious stages: tachyzoite, bradyzoite, and sporozoite, which share similar cellular structures. In our study, the tachyzoites were harvested from in vitro cultured human foreskin fibroblasts infected with *T. gondii* for cryo‐ET imaging (Figures S1 and S2, Supporting Information).

**Figure 1:**
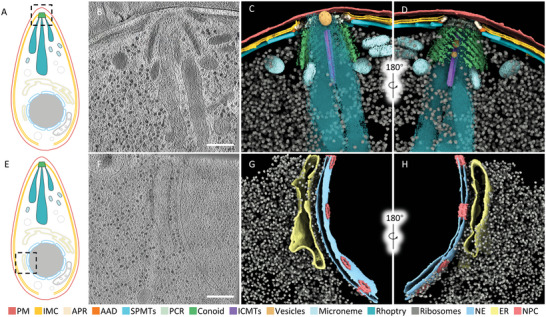
Overall architecture of the *T. gondii* tachyzoite. A,E) Schematic of a whole tachyzoite. The boxed regions were imaged with cryo‐ET. B,F) Tomographic slices of tomograms exhibit areas around B) the conoid and F) NE. Scale bars: 200 nm. C,G) 3D rendering of tomograms corresponding to the regions of (B) and (F). D,H) The back‐side view of (C) and (G). Visible subcellular structures were colored as indicated. (PM: plasma membrane; IMC: inner membrane complex; APR: apical polar ring; AAD: amorphous APR‐associated density; SPMTs: subpellicular microtubules; PCR: pre‐conoidal ring; ICMTs: intra‐conoidal microtubules; NE: nuclear envelope; ER: endoplasmic reticulum: NPC: nuclear pore complex).

We were able to identify several subcellular structures from the reconstructed tomograms (**Figure** **1**). The PM is the outermost monolayer structure of a tachyzoite. Beneath the PM are two closely apposed membranes, known as the IMC. The SPMTs, which start from the APR and extend toward the basal end, serve as the cytoskeleton to maintain the shape of cells underneath the IMC. The funnel‐shaped conoid is located at the apical end of the tachyzoite and protrudes from the APR during invasion.^[^ ^16^ ^]^ The pre‐conoidal ring (PCR) is an annular structure at the top of the conoid, and the ICMTs are a pair of parallel microtubules located in the center of the conoid. Additionally, secretion‐related structures including club‐shaped rhoptry and rod‐like micronemes were also observed (Figure 1A–D). The four‐layered membrane organelle apicoplast, a unique organelle in Apicomplexan parasites, was clearly found near the tachyzoite nucleus (Figure S3A, Supporting Information). Interestingly, the mitochondria contain spherical cristae (Figure S3B, Supporting Information). This may be a unique feature of this parasite, as it has a strong ability to adapt to warm‐blooded animals. Therefore, more work should be performed in the future to understand this finding. In addition to these organelles, putative protein complexes such as the ribosome, proteasome, and nuclear pore complex (NPC) were also identified and averaged (Figure 1E–H). Surprisingly, the cytoplasmic ring density of the putative NPC was largely missing in *T. gondii*. To exclude the possibility that this was caused by the image processing method or the limited data size, the NPC structure of mouse neutrophil was reconstructed using an identical protocol and a similar number of particles, and the cytoplasmic ring density was well resolved (Figure S3C, Supporting Information). This missing density most likely reflects the structural flexibility, while the role of this unique feature remains enigmatic.

### 2.2 Analysis of Microtubules in Tachyzoites

**Figure 2:**
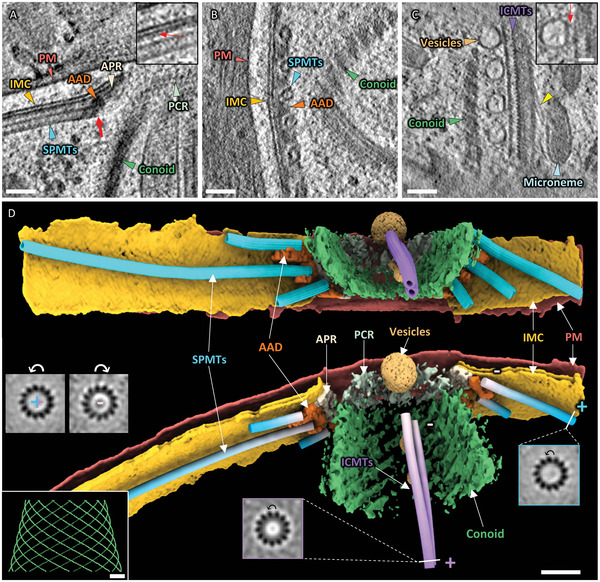
Distribution of SPMTs and ICMTs in the apical region of *T. gondii* tachyzoites. A) A tomogram slice highlights the end of an SPMT associated with AAD. The red arrow indicates the end of SPMT. In the inset, the red arrow indicates the density attached to the IMC between the IMC and SPMT. B) A tomogram slice displays the AAD caught between two adjacent SPMTs. C) A tomogram slice shows two apposed ICMTs. The left ICMT is associated with four vesicles, while the right one is arrayed with a hairy structure marked with a yellow arrowhead. The red arrow in the inset indicates densities tethering a vesicle to ICMT. D) 3D rendering of the apical region from two views. SPMTs and ICMTs are colored with blue and purple gradients with white and colored regions denoting plus and minus ends, respectively. The insets are cross‐sections of the averaging results from the microtubules. The “counter‐clockwise skew” indicates it was viewed from the plus end. The intact conoid model simulated using the density of the visible conoid fibers from this tomogram is shown in the bottom left corner. (PM: plasma membrane; IMC: inner membrane complex; APR: apical polar ring; AAD: amorphous APR‐associated density; SPMTs: subpellicular microtubules; PCR: pre‐conoidal ring; ICMTs: intra‐conoidal microtubules). Scale bars: 50 nm in (A–C) and inset of (D), 100 nm in (D), and 20 nm in the inset of (A) and (C).

Microtubules, a component of the cytoskeleton, play a vital role in cellular activities such as movement, transport, and division. A typical tachyzoite contains ≈22 SPMTs and 2 ICMTs.^[^ ^7^, ^11^, ^17^ ^]^ Our results largely agreed with these previous findings (**Figure** **2** and Movie S1, Supporting Information). The SPMTs underlie and extend along the surface of the IMC to maintain the shape of the tachyzoite, while the ICMTs start from the inner conoid and grow toward the basal end. The ICMTs, which are considered intracellular transportation scaffolds, are much shorter than the SPMTs and are attached to vesicles on one side.^[^ ^9^ ^]^

We observed that the density that connects the SPMTs to the APR is the amorphous density associated with APR (AAD) (Figure 2A), which we also observed between adjacent SPMTs (Figure 2B), and may function as a linker for the SPMTs, APR, and IMC.^[^ ^18^ ^]^ A linker between SPMTs and IMC was reported in *Plasmodium* sporozoites^[^ ^19^ ^]^ but was rarely observed in our investigation on tachyzoite of *T. gondii*, while a continuous layer of densities was found attaching to the IMC (Figure 2A inset). The AAD was only attached to one side of the end of the SPMTs, with very little evident density capping the end of the SPMTs. Therefore, the mechanism for the stability of this end is ambiguous. ICMTs are comprised of two closely apposed microtubules. One ICMT was arrayed with four vesicles via linkers, while the other was attached by hairy structures of unknown function (Figure 2C).

To resolve the delicate structures of the SPMTs and ICMTs, we traced along those microtubules and applied subtomogram averaging. Our results showed 13 protofilaments and an 8 nm repeat in the inner wall of both the SPMTs and ICMTs (Figure S4, Supporting Information), which is consistent with the length of a dimer of *α*‐ and *β*‐tubulin.^[^ ^20^ ^]^ This result indicates that the structure of the microtubule inner proteins (MIPs) of ICMTs is possibly similar to SPMTs. The polarity of the microtubules was determined by adapting a previously developed method.^[^ ^21^ ^]^ The minus ends of the SPMTs (*N* = 18) were close to the conoids, while the plus ends were oriented toward the basal direction of the tachyzoites (Figure 2D), which is consistent with the sporozoites of *Eimeria acervulina* and *E. tenella*.^[^ ^22^ ^]^ For the ICMTs (*N* = 13), the polarity analysis results showed an extension direction from the inner conoids toward the basal end of the tachyzoites, identical to that of SPMT (Figure 2D). The minus ends of the SPMTs and ICMTs were close to the APR in spatial distribution, supporting the potential role of the APR as a microtubule organization center.^[^ ^22^ ^]^ Collectively, these results indicate that microtubules play an essential function in maintaining the morphology of tachyzoites.

### 2.3 Structural Analysis of Daughter Buds in Tachyzoites

**Figure 3:**
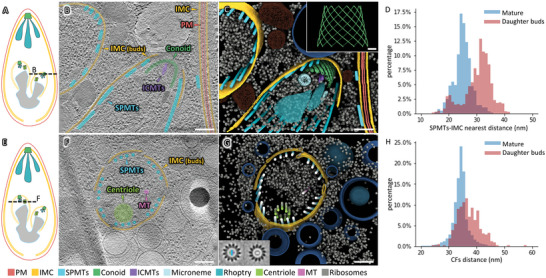
Annotation of daughter buds from different *T. gondii* mother cells. A,E) the cartoon depicts different parts of daughter buds within mother cells. The parts displayed in images (B) and (F) are from two different mother cells marked in the cartoon and indicated by dashed lines. B,F) Tomographic slices of tomograms exhibit daughter buds with B) conoid and F) centriole in the cytoplasm of mother cells. The IMC of the daughter buds was annotated with “IMC (buds).” C,G) 3D rendering of tomograms corresponding to regions (B) and (F). Other membranous organelles are labeled in navy blue. The inset in (C) displays an intact conoid model simulated using visible conoid fibers from this tomogram. The inset in (G) displays a cross‐section of two averaged microtubules, the “counter‐clockwise/clockwise skew” indicates it was viewed from the plus/minus end, respectively. D) Statistical distribution of the distance between SPMTs and the nearest IMC in mature tachyzoites (blue) or daughter buds (red). H) Statistical distribution of the distance between two adjacent conoid fibers in mature tachyzoites (blue) and daughter buds (red). A one‐sided Kolmogorov‐Smirnov test was applied, and the result showed *p*‐value < 0.01. (PM: plasma membrane; IMC: inner membrane complex; SPMTs: subpellicular microtubules; ICMTs: intra‐conoidal microtubules; MT, microtubule). Scale bars: 200 nm in (B), (C), (F), and (G). 50 nm in the inset of (C).

The asexual reproduction of *T. gondii* occurs primarily by endodyogeny, one of the characteristics of Apicomplexan parasites. In this process, two daughter buds are generated inside a mother cell.^[^ ^12^ ^]^ In our data, we observed several tomograms with daughter buds (*N* = 10) in the cytoplasm of mother cells. To uncover the structural changes of the cytoskeleton during this process, we annotated the subcellular structures of the daughter buds and compared them with those from the mature tachyzoites (**Figure** **3**A–C,E–G and Movie S2, Supporting Information).

Identical to mature tachyzoites, underneath the IMC, the SPMTs exist in daughter buds to maintain their shape (Figure 3A–C,E–G). To further analyze the interaction between the SPMTs and IMC, we calculated the distance between them in both mature tachyzoites and daughter buds. We found that the space was ≈25 nm in mature tachyzoites, while more extensive and varied in daughter buds (Figure 3D). This difference may be caused either by the effect of the iso‐osmotic environment, or by the benefits to the daughter buds for cell volume enlargement that may result from a weaker interaction.

In a cross‐section view of the daughter bud, we observed 22 SPMTs (Figure 3F) with identical polarities toward the basal direction (Figure 3G), which are consistent with those found in mature tachyzoites. Surprisingly, the SPMTs were not evenly spaced but arranged in a “4 + 4 + 4 + 4 + 4 + 2” pattern (Figure 3F,G), with larger inter‐group spacing than intra‐group spacing. Interestingly, the inter‐group spaces corresponded to the gaps between the alveoli of the IMC, suggesting that the uneven distribution of SPMTs may be relevant to IMC expansion.

It is well known that the centriole plays a vital role in cell reproduction.^[^ ^13^ ^]^ Interestingly, we found that the centriole of *T. gondii* was much smaller than that in humans and consisted of singlets rather than triplets (Figure S5, Supporting Information). According to our 3D segmentation, the singlets were not strictly parallel to each other (Figure S5C, Supporting Information). Like all other microtubules, these singlets were formed by 13 protofilaments. Although we have no idea so far how the progress of the protofilaments occurs, this novel finding raised an interesting question about how microtubules determine their destination. In contrast to other species, relatively few free microtubules were found around the centriole (Figure S5A,B, Supporting Information), suggesting that the role of the centriole during reproduction in this fascinating organism is something other than the microtubule‐organizing center (MTOC).

### 2.4 The Conoid Fiber of *T. Gondii* May Be Derived from the Microtubule

In *T. gondii*, the conoid is located in the apical end and possesses both “protruded” and “retracted” states, which is a typical characteristic of Apicomplexan parasites. During the invasion process, which is activated by calcium flux, the conoid protrudes from the APR, and proteins from rhoptries and micronemes are secreted to induce internalization.^[^ ^8^ ^]^ The shape of an intact conoid is a funnel comprised of many spiral conoid fibers forming a spring‐like structure (Figure 2D). The conoid fibers are mainly comprised of tubulins, and their cross‐sections display a comma shape, which differs from microtubules.^[^ ^23^ ^]^

**Figure 4:**
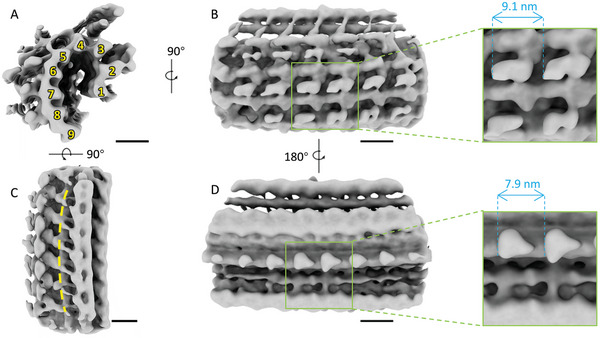
Structural analysis of the *T. gondii* conoid fiber in the retracted state. A) Cross‐section of the conoid fiber segment. Nine tubulin protofilaments are arranged into a comma shape, as indicated by the yellow numbers. B–D) Density map of the conoid fiber segment from different views as indicated. A representative repeated patch is marked with a green box, and patch size is measured from both the B) convex side and the D) concave side. The yellow dashed line along the segment indicates the curved structure (C). Scale bars: 10 nm.

In total, we identified 13 conoids in the mature tachyzoite, of which 10 were in the retracted state; the remaining conoids were indistinguishable due to the incomplete cellular context resulting from the cryo‐FIB microfabrication. This ratio of conoid states observed in our work strongly suggests that conoids tend to retract when the tachyzoite is not undergoing the invasion process. This function may accord with the principle of muscle contraction in metazoa and is the best way to save energy. In addition, our results support the fact that the conoid protrudes from the APR, and that proteins from rhoptries and micronemes are secreted only during the invasion process.^[^ ^8^ ^]^ For the mature tachyzoite, the overall shape of the conoids we measured (*N* = 8) was consistent with the results from different laboratories:^[^ ^11^, ^18^ ^]^ a funnel‐like shape ≈250 nm in height and 265–375 nm in diameter (Figure 2D). The structure of the conoid fibers was resolved at 28 Å by averaging 1082 particles (**Figure** **4**). The backbone of the structure was formed from nine protofilaments potentially made of tubulin (Figure 4A). The structure showed a noticeable repeat feature with a patch size of ≈9.1 nm on the convex surface and ≈7.9 nm on the concave surface due to the curvature of the conoid fibers (Figure 4B–D), which are close to the length of *α*‐ and *β*‐tubulin dimers. Due to the conoid's cone shape, the conoid fibers' curvature is different in the apical and basal parts of the conoid, limiting the resolution of the averaging result.

**Figure 5:**
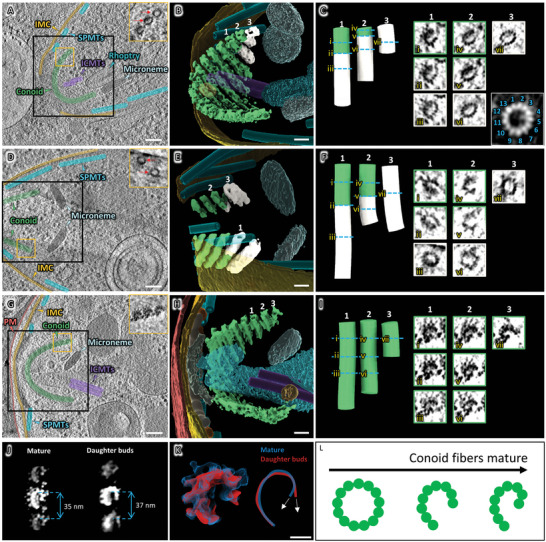
Structural differences of conoids between daughter buds and mature tachyzoites in *T. gondii*. A,D,G) Tomographic slices of A,D) daughter buds and G) a mature tachyzoite with conoid. The areas (within the yellow box) with conoid fibers are shown in the insets. The circular conoid fibers are indicated by red arrows. B,E,H) 3D rendering of tomograms highlighting the areas with the conoid [black boxes indicated in (A), (D), and (G)]. C,F,I) Representative conoid fibers labeled in (B), (E), and (H) are shown as cartoon surfaces. The conoid fibers were colored depending on their cross‐sectional shape: white for the ring shape and green for the comma shape. Cross‐sections (≈ 20 nm thick) of selected regions were shown as indicated by Roman numerals. The cross‐section of the averaged structure of the ring shape region was shown as an inset in (C), with protofilaments denoted. J) 2D projection of averaged conoid fibers without masking away the adjacent densities. The distances between adjacent fibers were measured and annotated. K) Left: cross‐section of averaged conoid fibers structures from mature tachyzoites (blue) and daughter buds (red). Right: profile lines indicate the protofilament arrangement of conoid fibers from mature tachyzoites (blue) and daughter buds (red) by tracing the averaged structures shown on the left. The two white arrows indicate the difference in curvature. L) Schematic representation of the maturation process of conoid fibers. Green circles indicate the protofilaments. (PM: plasma membrane; IMC: inner membrane complex; SPMTs: subpellicular microtubules; ICMTs: intra‐conoidal microtubules). Scale bars: 100 nm in (A), (D), and (G); 50 nm in (B), (E), and (H); 10 nm in (K).

In the apical part of the daughter bud, the conoid was visible under a retracted state, with the overall architecture similar to that of the mature tachyzoites (Figure 3C and **Figure** **5**A,B,D,E,G,H). Compared to the mature tachyzoites, the overall structure in the daughter buds was less compact, with looser spacing between adjacent conoid fibers (Figures 3H and 5J). These differences indicate that the conoid is likely undergoing maturation, which involves morphological changes and topological packing of the conoid fibers.

To validate this finding, we focused on the structural details of the daughter bud's conoid fiber. Surprisingly, the cross‐section of some conoid fibers showed a circle shape while others were a typical comma shape (Figure 5A,D inset). The results from both the 2D projection classification and 3D reconstruction of the circle shape regions showed a clear hollow tube with a 26 nm diameter made up of 13 protofilaments, reminiscent of the microtubule structure (Figure 5C inset). The comma‐shaped fibers were analyzed as mature tachyzoites; limited by particle number and structural heterogeneity, an average map with a moderate resolution was achieved, which showed a less compact shape than the mature tachyzoite (Figure 5K). We further found that the circle shape and comma shape cross‐sections co‐existed in the same conoid fiber with a clear transition zone (Figure 5B,C,E,F). The circle shape form was found only at the basal end of the conoid, which gradually transformed to the typical comma shape form along the conoid fiber. However, because the intact conoid was truncated during cryo‐FIB fabrication, we were unable to determine the exact position of the transition for each conoid fiber.

In contrast, all conoid fibers were in the comma shape form in the mature tachyzoite (Figure 5G–I). These results suggest that the conoid of the daughter bud is in a process of maturation, with the maturation direction from the apical to the basal end. Considering that the backbone of the conoid fiber is composed of tubulin and that the microtubule is one of the most tubulin‐enriched structures, we suggest that the tubulin of the conoid fiber may originate from microtubules. During this process, the conoid fibers were transformed from a hollow tube to a comma‐shaped fiber, losing 4 of the 13 protofilaments to reach the mature form (Figure 5L). This transformation process is reasonable since the stiffness of hollow microtubules prevents the formation of a highly curved conoid. This transformation takes place within a range of about 10 nm. However, the available data is insufficient to determine if there is an incremental maturation process.

## 3 Summary

In summary, we determined the molecular anatomy of *T. gondii* tachyzoites at nanometer resolution using state‐of‐the‐art cryo‐ET technology, with a particular focus on the tubulin‐based cytoskeleton. Our study not only confirmed the results published, but also presented interesting novel features. Importantly, among them, we captured the structural details of daughter buds in 3D for the first time, highlighting the potential microtubule origin of the conoid fiber, which is the critical component of the secretory system. A better understanding of this process will provide a new approach for regulating *T. gondii* reproduction, which may serve as a novel drug target. Currently, our findings were limited by resolution and data size; therefore, we were unable to reveal the full details of this regulatory mechanism. Using correlative electron light microscopy, tachyzoites undergoing endodyogenesis could be the target of future studies to more efficiently capture images of daughter buds. Furthermore, with CRISPR technologies, the genetic screening of *T. gondii* is possible.^[^ ^24^ ^]^ The use of loss‐of‐function analyses provides hope that the critical components of this process can be identified in the near future.

It is also notable that, not only is the potential origin of conoid fibers identified, but also all microtubules in *T. gondii* are shown to contain 13 protofilaments, suggesting a common packing of tubulins. However, the differing subcellular destinations and functions of these cytoskeleton components highlight the need for future research to focus on their regulation.

## 4 Experimental Section

### Culture and Maintenance of *T. gondii*

The *T. gondii* RH strain was used in this work. It was maintained in one of the authors’ laboratory (ZRL, SYSU). To culture this parasite, the frozen tachyzoites in liquid nitrogen were thawed and added to human foreskin fibroblasts (HFFs) cultured with Dulbecco's modified Eagle's medium (DMEM). The medium was changed every 12–24 h depending on the goal of the experiments.

To harvest the tachyzoites, the infected HFFs were digested using trypsin and passed through a 27‐gauge syringe several times to mechanically disrupt the HFFs and release the tachyzoites. To remove the disrupted HFFs, the suspension was filtered with a 5 µm sterile syringe filter. To collect the tachyzoites, the filtered suspension was centrifuged for 10 min at 1000 *g*. The supernatant was removed, and the tachyzoites were resuspended in DMEM (with 30% FBS and 10% DMSO) for storage or vitrification.

### Cryo‐ET Sample Preparation

The resuspended tachyzoites described above were loaded onto R 2/1 EM grids (Quantifoil, Jena, Germany), which were freshly glow discharged in a Model 950 Advanced Plasma System (GATAN, Pleasanton, CA, USA) before use. Excess liquid was blotted away from the back, and grids were plunge‐vitrified with the Vitrobot Mark IV system (Thermo Fisher Scientific, Waltham, MA, USA). The vitrified samples were stored in liquid nitrogen until milling.

### Cryo‐FIB Milling

To prepare lamellae for cryo‐ET, cryo‐FIB milling was performed following a previous protocol (Figure S2A–C, Supporting Information).^[^ ^25^ ^]^ Briefly, the vitrified samples were loaded into the Aquilos 2 Cryo‐FIB system using a cryo‐transfer rod (Thermo Fisher Scientific). To reduce the accumulation of electrons on samples and provide extra support while milling, sputtering was first performed on the samples with a 30 mA current for 15 s followed by organometallic platinum (Pt) deposition via GIS for 20 s. A 500 pA current was used for rough milling around the target region, and then the current was reduced gradually to 10 pA for fine milling. After the thickness of the lamellae reached ≈200 nm (Figure S2C, Supporting Information), Pt sputtering was performed again using a 7 mA current for 7 s to prevent charging during tilt series collection.

### Tomographic Tilt Series Collection

The milled lamellae were loaded into a 300 kV Titan Krios transmission electron microscope equipped with a K3 camera and Gatan energy filter (Figure S2D, Supporting Information). Tilt series were automatically collected using SerialEM software.^[^ ^26^ ^]^ Images were acquired using super‐resolution mode resulting in 10 frames per image, and the magnification was set at 26 000× (pixel size was 3.328 Å), and the defocus was set between −3.0 and −5.0 µm. The tilt series were collected from −60° to 60° (relative to the pre‐tilt angle) in 3° increments and the bidirectional acquisition scheme was applied.^[^ ^27^ ^]^ The total dose was limited to ≈110 e^‐^ Å^−2^ for each tilt series.

### Tomogram Reconstruction and Membrane Segmentation

The frames of each tilt were motion‐corrected using MotionCor2 software,^[^ ^28^ ^]^ and merged into tilt series. The tilt series were reconstructed in IMOD software using the patch‐tracking and back projection method (Figure S2E, Supporting Information).^[^ ^29^ ^]^ For visualization and segmentation, the resulting tomograms were downscaled using a binning factor of 4 or 6, and deconvolution was performed in MATLAB R2019b to improve the contrast.^[^ ^30^ ^]^

The membrane segmentation was performed using a tensor voting‐based method,^[^ ^31^ ^]^ and the results were then polished manually in Amira 2020.3.1.^[^ ^32^ ^]^ ChimeraX 1.3^[^ ^33^ ^]^ was used to render the segmentation results.

### Microtubules

A total of 142 microtubules (including SPMTs and ICMTs) were manually traced in the tomograms using IMOD 4.11.0 as described.^[^ ^29^ ^]^ To determine the polarity of each microtubule, alignment, and averaging were performed for each microtubule. To reduce the effect of the missing wedge, helical symmetry was applied: it was speculated that the rise and twist of microtubules were 0.92 nm and 27.69°, respectively, since the majority of microtubules in *T. gondii* contained 13 pfs.^[^ ^11^, ^34^ ^]^ Particles (6× binned, 19.968 Å pixel^−1^) were cropped with a box size of 24 pixels^3^ and aligned in Dynamo 1.1.532 software^[^ ^35^ ^]^ using the helical parameter mentioned above. Additionally, the “cone aperture” and “azymuth rotation range” were restricted to 60° and 28°, respectively. The averaging results of each microtubule's cross‐section displayed a “clockwise skew” if viewed from the minus end. After unifying the polarity, particles (1× binned) were merged and 3D refinement was applied for SPMTs and ICMTs separately. As helical symmetry was applied, the “seam” of the microtubule would be altered and the *α*/*β*‐tubulins would be indistinguishable. Other helical parameters were tried for alignment, but no clear average was reached, indicating that all the microtubules contain 13 pfs.

### Conoid

To unify the polarity of the conoid fibers, the fibers were manually traced from the apical to the basal ends in Chimera 1.15 software^[^ ^36^ ^]^ using “Volume Tracer.” A total of 1082 particles (1× binned) with a box size of 204 pixels^3^ were generated by cropping along the conoid fibers with 17 nm space and then aligned in RELION 2.1 software.^[^ ^37^ ^]^ The result was imported into M 1.0.9 software^[^ ^38^ ^]^ and refined with a soft mask to improve resolution. The final resolution was 28 Å.

To obtain the structure of the conoid fibers in the daughter buds, the conoid fibers were manually traced using the same method described above. The particles were cropped with 8 nm space according to the averaged result in the mature tachyzoites. The 2D classification was applied to separate conoid fibers under different maturation statuses. A circle shape 2D average with 13 pfs was reached. However, limited by particle number, no meaningful 3D structure could be resolved for these particles. The remaining 290 particles were averaged in 3D to get the structure of the comma‐shaped conoid fiber with a resolution of 37 Å. The distance between adjacent conoid fibers was measured in the cross‐section view of the averaged structure.

To simulate the intact conoid via incomplete density of the conoid in tomograms, it was surmised that the overall shape of the conoid was the “frustum of a cone.” The “frustum of a cone” model, created in MATLAB with a height of 250 nm and a diameter of 265 nm (apical end) and 375 nm (basal end), was able to adequately fit the conoids of the mature tachyzoites in the tomograms. The number and twist angle of the fibers varied slightly among the different conoids. The conoid in the daughter bud was simulated via the same method with different “frustum of a cone” parameters (height: 250 nm; diameter of the apical end: 210 nm; diameter of the basal end: 300 nm).

### Centriole

For *T. gondii*, the centriole microtubule singlets were automatically traced in Amira. The particles were cropped in Dynamo 1.1.532, and the helical symmetry was applied as previously described for averaging the microtubules. The generated 1393 particles (1× binned) with a box size of 192 pixels^3^ were aligned in Dynamo and refined in RELION 2.1.

For the Hela cells, the data was collected in a pixel size of 4.3 Å pixel^−1^, and the centriole microtubule triplets were traced manually along the B‐tubules in IMOD 4.11.0. The particles were cropped every 8 nm, resulting in 339 subtomograms with a box size of 168 pixels^3^ (2× binned) for alignment in RELION 2.1.

### NPC

NPC particles with integrity of more than half were picked manually using IMOD 4.11.0. A total of 62 particles (2× binned) in the tomograms of *T. gondii* were aligned in RELION 2.1 and C8 symmetry was applied. The tilt and psi were restricted so that the CR of the NPC would not be aligned to the NR. The resolution of this structure was ≈67 Å. To test the robustness of the pipeline, the same method was applied to obtain the mouse NPC structure using a neutrophil sample from the lab. A density map with a resolution of 62 Å was obtained by averaging 40 particles.

### Distance between SPMTs and the Nearest IMC

The distance between SPMTs and the nearest IMC was calculated using scripts from the previous study^[^ ^39^ ^]^ with modifications. In brief, the IMC was segmented manually in Amira, while the SPMTs were traced using the coordinates obtained from the results of the 3D refinement. The distances between points (along SPMTs) and the IMC were counted as the distance between the SPMTs and IMC. A total of 30 SPMTs in mature tachyzoites and 25 SPMTs in daughter cells were used for statistics, and a one‐sided Kolmogorov‐Smirnov test was used in Python 3.8.0 to determine statistical significance.

### Distance between Adjacent Conoid Fibers

The conoid fibers were traced using the coordinates obtained from the results of the 3D refinement, and the distance of points between adjacent conoid fibers was calculated. A total of 76 adjacent conoid fibers in mature tachyzoites and 9 adjacent conoid fibers in daughter buds were counted. A one‐sided Kolmogorov‐Smirnov test was used in Python to determine statistical significance.

## Conflict of Interest

The authors declare no conflict of interest.

## Supporting information

Supporting Information

Supplemental Movie 1

Supplemental Movie 2

## Data Availability

Two representative tomograms have been deposited in the Electron Microscopy Data Bank (EMDB) as EMD‐35097 and EMD‐35098. The conoid fiber average has been deposited in the EMDB as EMD‐35096.
