# Supplementary material for: Cryo‐Electron Tomography of Toxoplasma gondii Indicates That the Conoid Fiber May Be Derived from Microtubules
Source: Adv Sci (Weinh). 2023 Feb 25;10(14):2206595. doi: 10.1002/advs.202206595 (PMC10190553; doi:10.1002/advs.202206595)
Supplement: Supplementary file 1 — Supporting Information [file ADVS-10-2206595-s003.pdf]

Supporting Information

Cryo-electron tomography of *Toxoplasma gondii* indicates that the conoid fiber may be derived from microtubules

Zhixun Li[1], Wenjing Du[1], Jiong Yang[2], De-Hua Lai[2], Zhao-Rong Lun[2] , Qiang Guo[1,3,*]

1. State Key Laboratory of Protein and Plant Gene Research, Peking-Tsinghua Center for Life Sciences, Academy for Advanced Interdisciplinary Studies, School of Life Sciences, Peking University, Beijing 100871, China

2. State Key Laboratory of Biocontrol, School of Life Sciences, Sun Yat-Sen University, Guangzhou 510275, China

3. Changping Laboratory, Beijing, China

* Corresponding author: Qiang Guo, guo.qiang@pku.edu.cn

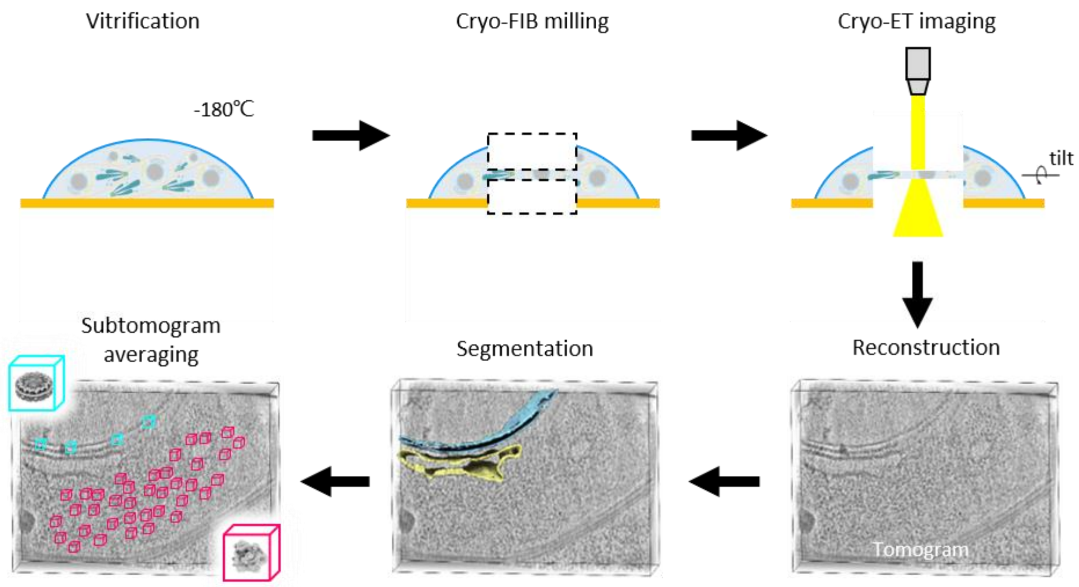

Figure S1 Flow chart of the cryo-ET workflow process of T. gondii. *The tachyzoites suspension was loaded onto a glow-discharged grid and vitrified at -180 ℃ (vitrification). Then cryo-FIB was applied to mill the vitrified sample (cryo-FIB milling). Afterward, the milled lamella was tilted and imaged in cryo-EM (cryo-ET imaging). The collected tilt series images were reconstructed to generate a tomogram (reconstruction). 3D segmentation was performed for subcellular structures in the tomogram (segmentation). Subtomogram averaging was applied for macromolecules in the tomogram to generate an averaged structure with higher resolution (subtomogram averaging).*

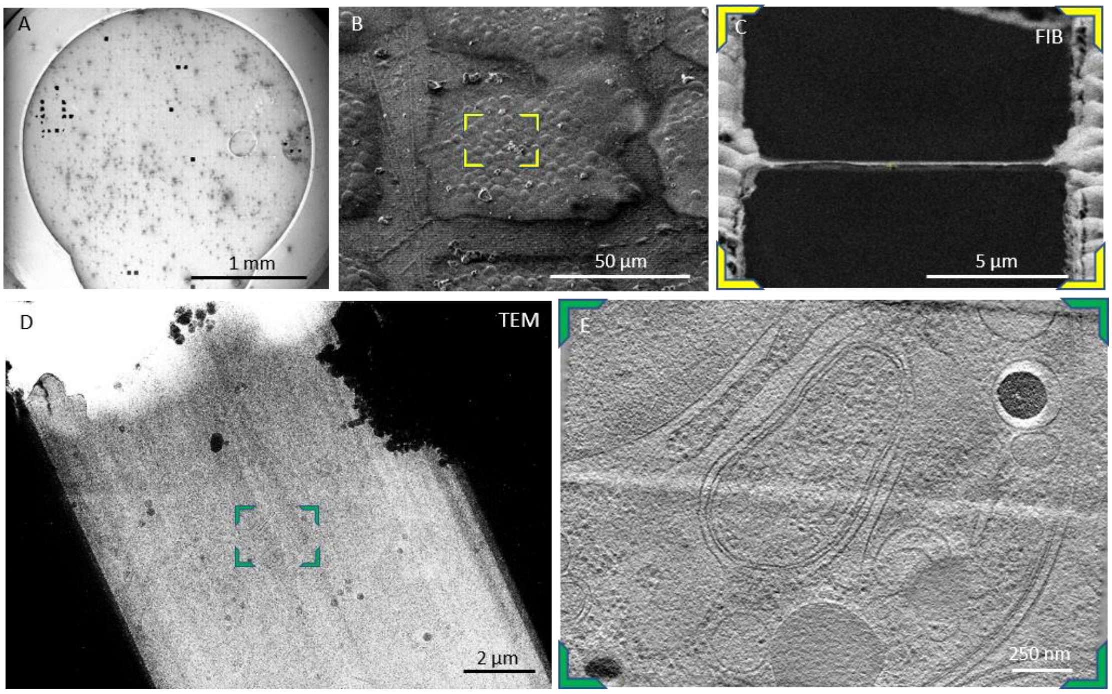

Figure S2 Cryo-ET process for T. gondii tachyzoites. A*, The vitrified sample was loaded into the cryo-FIB system and coated with Pt.* ***B****, The sample was imaged in SEM view, and a yellow box marked the target region for milling.* ***C****, The target region marked in* ***B*** *was milled and imaged using an ion beam.* ***D****, The milled lamella was imaged in a Titan Krios 300 kV cryo-TEM, with the target region marked with a green box imaged on a higher magnification.* ***E****, A slice of tomogram reconstructed using the tilt series images collected from the target region. (TEM: transmission electron microscope).*

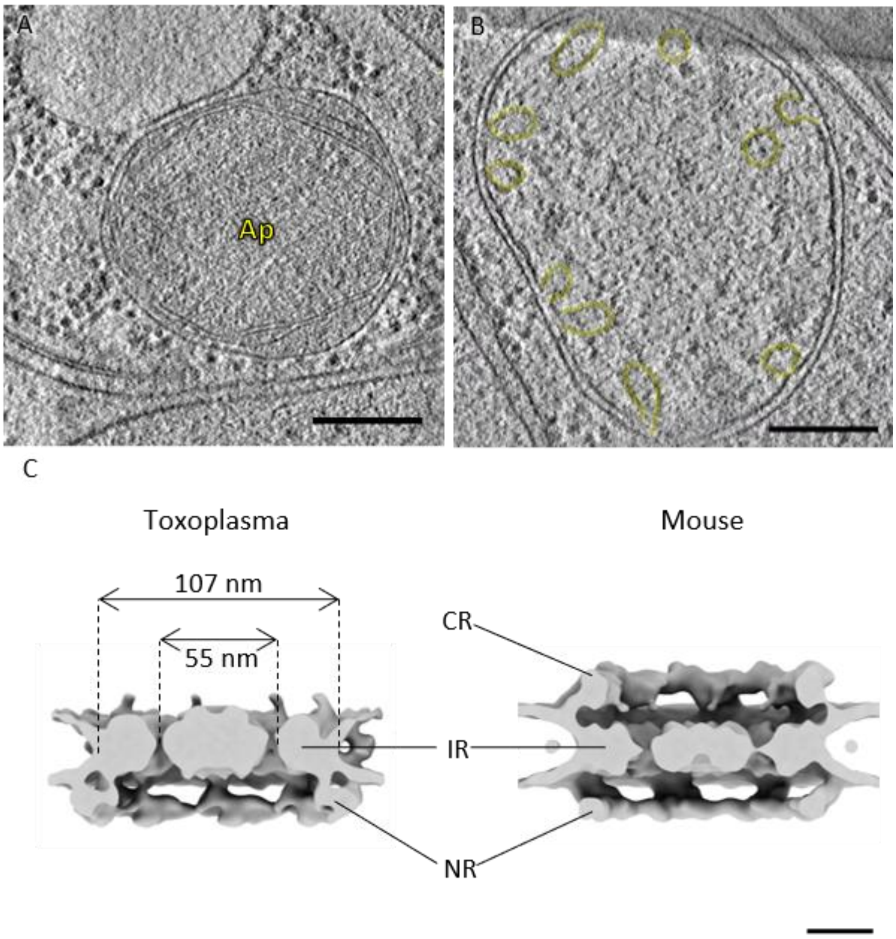

Figure S3 Annotation of the apicoplast and mitochondria and the in situ structure of NPC. ***A***, *A tomogram slice shows an apicoplast in tachyzoite. Multiple layers of membranes are visible, which is consistent with previous descriptions.* ***B***, *A tomographic slice displays a mitochondrion's spherical cristae (indicated by yellow shadows).* ***C***, *Density maps of the NPC of T. gondii (left) and mouse (right) are shown from the cut-off view. (Ap: apicoplast; CR: cytoplasmic ring, IR: inner ring, NR: nuclear ring). Scale bars: 200 nm in* ***A*** *and* ***B***, *30 nm in* ***C***.

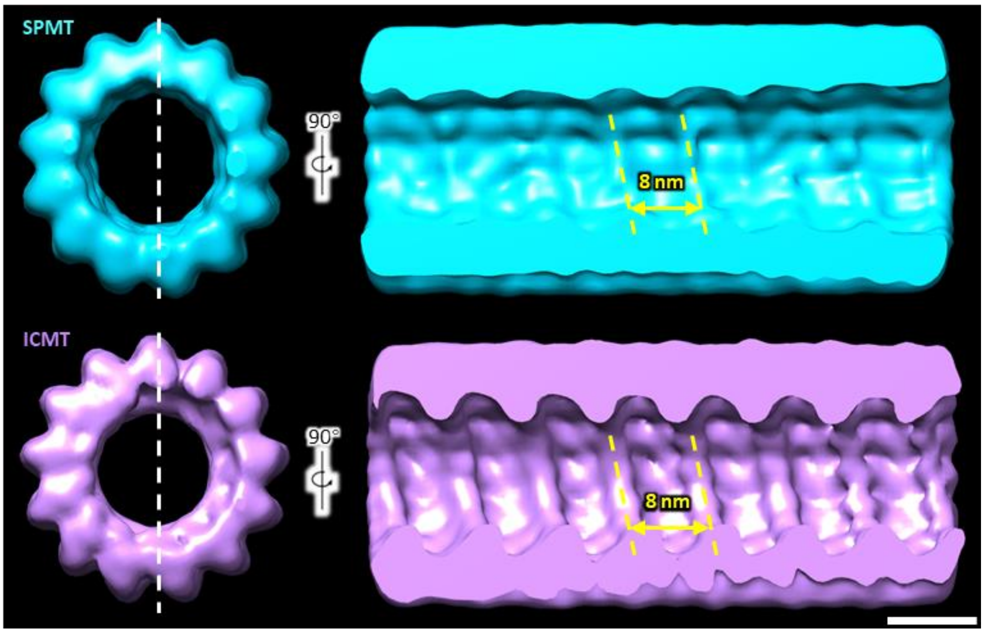

Figure S4 Averaged structures of SPMTs and ICMTs in T. gondii tachyzoites. *The averaged structure (left) and its cross-section view (right) of SPMT (top) and ICMT (bottom) are shown. (SPMT: subpellicular microtubules; ICMT: intra-conoidal microtubules). Scale bar: 10 nm.*

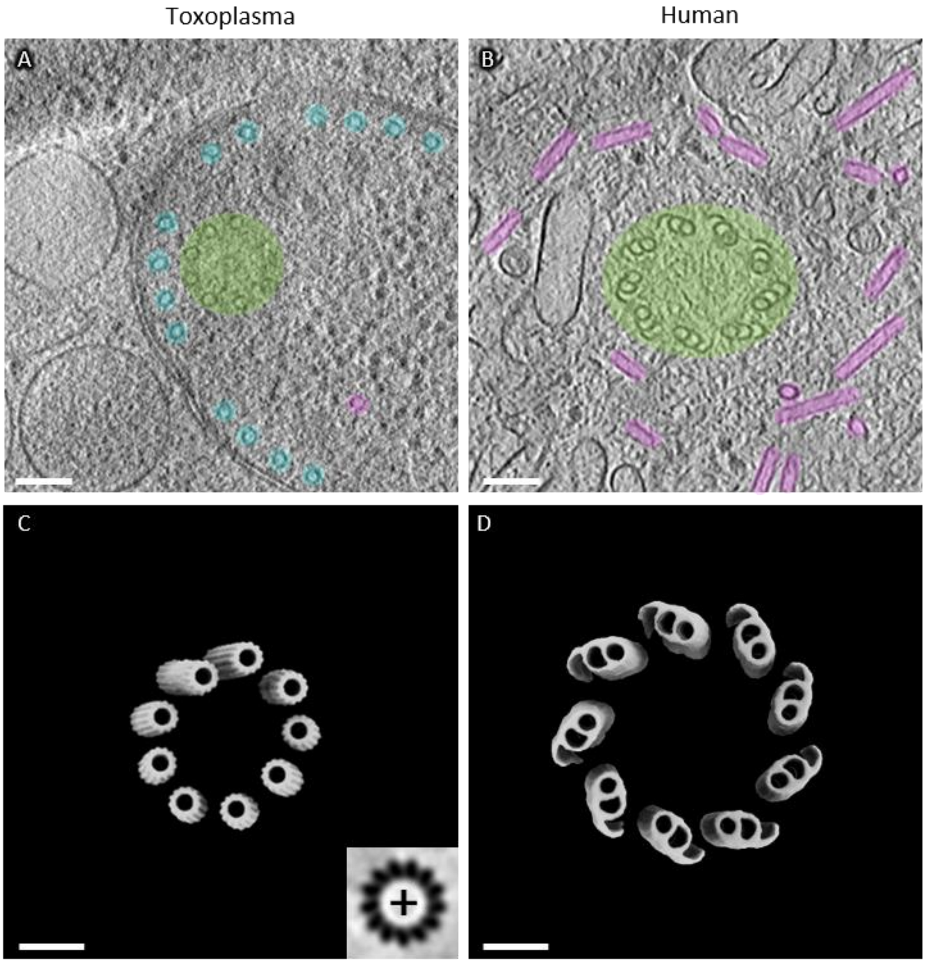

***Figure S5 Comparison of centrioles in T. gondii and human cells. A** and **B**, Slices of tomograms display the centrioles in the native environment from T. gondii (A) and human cells (B, EMD-33495). Green shadows indicate the centrioles, SPMTs are indicated by blue shadows, and other microtubules are indicated by purple shadows. **C** and **D**, 3D rendering of centrioles from T. gondii (C) and human (D) cells in the top view. (SPMTs: subpellicular microtubules). Scale bars: 100 nm in **A** and **B**, 50 nm in **C** and **D**.*

Movie S1 Molecular annotation of T. gondii tachyzoite apical region

Movie S2 Molecular annotation of T. gondii daughter bud apical region
